# Causal relationship of garlic or onion with gastric cancer based on a Mendelian randomization study

**DOI:** 10.1097/MD.0000000000041639

**Published:** 2025-05-02

**Authors:** Zhaoyin Wang, Pengfei Liu, Jingbin Wang, Pengli Ma, Xinyao Liu

**Affiliations:** a Heilongjiang University of Chinese Medicine, Harbin, Heilongjiang Province, China; b Department of Spleen and Stomach Diseases, Shenzhen Hospital (Fu Tian) of Guangzhou University of Chinese Medicine, Shenzhen, Guangdong, China.

**Keywords:** garlic, gastric cancer (GC), genome-wide association studies (GWAS), Mendelian randomization (MR), onion, single nucleotide polymorphisms (SNPs)

## Abstract

In observational studies, it has been known that garlic or onions have a negative causal relationship with gastric cancer (GC). In this study, we aim to explore the negative causal relationship between garlic or onion and GC through Mendelian randomization (MR) analysis. The instrumental variable selection for MR analysis is single nucleotide polymorphisms associated with garlic or onion, mainly using the inverse variance weighted method, combined with MR Egger, weighted media, simple mode, and weighted modes to evaluate their causal impact on GC. In addition, sensitivity analysis such as Cochran *Q* test, pleiotropy test, and leave-one-out method were used to evaluate the robustness of the impact of these single nucleotide polymorphisms on GC. The inverse variance weighted method showed a negative correlation between garlic and GC risk (odds ratio = 0.70; 95% confidence interval 0.49–0.99; *P* = .046), while there was no relationship between onion and GC, and the sensitivity analysis results showed robustness. The current study has revealed that garlic may be a factor in reducing the risk of GC, providing a strategy for preventing and treating GC.

## 1. Introduction

Gastric cancer (GC) is a common cancer with high incidence rate and mortality. GC ranks 5th in cancer incidence and fourth in cancer mortality. There are approximately 1.089 million gastric cancer patients worldwide, with a mortality rate of 769,000. This is equivalent to approximately 7 deaths per 10 gastric cancer patients, and a 5-year survival rate of 30%.^[1,2]^ According to the growth trend in 2020, by 2040, there will be 1.8 million new gastric cancer patients and 1.3 million new gastric cancer deaths per year, and the global burden of gastric cancer diseases will be 70% higher than in 2020.^[3]^ Therefore, exploring the prevention and treatment of gastric cancer has become a major global focus.

Some known risk factors for gastric cancer include environment, diet, and genetics. The main environmental risk factor is *Helicobacter pylori*, which is recognized as the main cause of gastric cancer.^[1,4,5]^ Dietary risk factors include alcohol, processed foods, high salt intake, high fat intake, animal based foods (meat, eggs, and dairy products), overeating, and irritating foods.^[5–7]^ The genetic risk factor is a family history of gastric cancer.^[8]^ Obviously, in addition to genetic factors, people can reduce the incidence of gastric cancer by improving environmental and dietary factors. Some studies have shown that fresh fruits and vegetables in the diet can reduce GC risk.^[9–11]^

Garlic and onions in the onion family vegetables, not only have edible and seasoning properties, but also possess antibacterial, antioxidant, and immunomodulatory properties, making them potentially anti-tumor and preventive against some cancers.^[12–17]^ It has been proven that various bioactive compounds such as allicin and onion A, which contain organic sulfur compounds in garlic and onions, have many potential anti-cancer mechanisms, including inhibiting cell proliferation, altering enzyme activity, and immune regulation.^[18–21]^ The active ingredients in garlic oil selectively increase the redox stress of cancer cells, leading to cell apoptosis and death^[22]^; extract of onion: bioactive steroid saponins (A-24) can induce tumor cell apoptosis and autophagy by regulating the oncogenic gene p53.^[23–26]^ Some clinical case–control observation studies have shown a significant negative correlation between garlic and onion vegetables in the onion family and GC.^[27–30]^ However, some observational studies have found no significant causal relationship between garlic or onion vegetables and GC.^[31,32]^ In summary, the evidence for the relationship between onion vegetables and GC risk is contradictory. Therefore, it is crucial to study the causal relationship between onion vegetables and GC.

Mendelian randomization (MR) is a new statistical method for studying the relationship between exposure and outcomes. The design principle of MR is to use exposure related heritable single nucleotide polymorphisms (SNPs) as instrumental variables (IVs) to evaluate the causal relationship between exposure and outcomes.^[33]^ Because genetic variation is randomly assigned during gamete formation and conception, SNPs are not affected by confounding factors, allowing the causal relationship evaluated by MR to avoid the influence of reverse causality and confounding bias.^[34]^ In this study, we used MR methods to investigate the causal relationship between garlic and onion and GC based on genome-wide association studies (GWAS) data.

## 2. Methods

### 2.1. Study design

This study strictly followed 3 key assumptions of MR method analysis: (1) the selected genetic IVs were significantly correlated with exposure factors (garlic or onion); (2) IVs were not associated with any other confounders; (3) the selected SNPs can only affect the outcome GC through exposure factors (garlic or onion), but not through other ways. If designed according to these 3 hypotheses, MR analysis can study the causal relationship between exposure factors (garlic or onion) and outcome (GC), while no confounding factors affect the analysis results.^[34]^

### 2.2. Data

The data used in this study were from the IEU opengwas database, which can be available online (https://gwas.mrcieu.ac.uk/). The details of the exposure (garlic or onion) and outcome (GC) datasets are shown in Table 1, and all samples were of European descent. More detailed information about GC can be obtained in the original study.^[35]^

**Table 1:** The garlic, onion, and GC GWAS summary-level data characteristics.

GWAS ID	Exposure/outcome	Sample size	Number of SNPs	ncase	ncontrol
ukb-b-17,223	Onion intake	64,949	9,851,867		
ukb-b-8124	Garlic intake	64,949	9,851,867		
ebi-a-GCST90018849	Gastric cancer	476,116	24,188,662	1029	475,087

### 2.3. Selection of IVs

First, this study used a significance threshold of *P* < 5 × 10^-5,[36]^ and screened out heritage IVs closely related to exposure factors (garlic or onion) in garlic or onion dataset. Next, this study set *R*^2^ < 0.001 and kb = 10,000 to remove linkage disequilibrium between IVs.^[37]^ Finally, this study set the F-statistic > 10 to eliminate weak IVs, and obtained reliable genetic variables.^[38]^

### 2.4. Statistical analysis

In this study, 5 MR methods including inverse variance weighted (IVW), MR-Egger, weighted median, simple mode, and weighted mode were used to analyze the causal effect of garlic or onion on GC. IVW regression does not consider the existence of the intercept term and uses the inverse of the outcome variance as the weight to fit. It is the standard, accurate, and robust standard for MR inference.^[39]^ Therefore, it is the primary analysis method of this study.

### 2.5. Sensitivity analysis

Sensitivity analysis mainly used horizontal pleiotropy test, heterogeneity test, and leave-one-out method. The MR-Egger regression method was used for the horizontal pleiotropy test.^[37]^ The intercept term in the MR Egger regression can be used to detect pleiotropy. If the intercept is significantly different from 0, it suggests the presence of pleiotropy.^[37]^ Cochran *Q* test method was used as the method of heterogeneity test in order to evaluate whether the 2nd and 3rd assumptions of MR analysis were met. In order to evaluate the robustness of the results, we adopt the leave-one-out method, which is to perform IVW analysis on the remaining SNPs after gradually eliminating each SNP, and compare the results with the original results.

## 3. Results

### 3.1. Selection results of IVs

According to the above screening method of IVs, the required IVs were selected. Firstly, a significance threshold of *P* < 5 × 10⁻⁵ was used to screen out genetic IVs closely related to the exposure factors of garlic or onion. Then, *R*² < 0.001 and kb = 10,000 were set to remove the linkage disequilibrium between IVs. Finally, F-statistic > 10 was set to eliminate weak IVs. This series of screening processes ensured that the selected SNPs could accurately reflect the relationship between the intake of garlic and onion and the risk of GC, and their strong association provided a reliable basis for the subsequent causal analysis. A total of 34 effective SNPs were included in the MR analysis of garlic or onion and GC risk (Table 2), including 19 effective SNPs for garlic and 14 effective SNPs for onion. The minimum value of their F value was 20.9, which indicated that SNPs were strong IVs.

**Table 2:** Instrumental variable characteristics of garlic or onion.

Exposure	SNP	EA	OA	Beta	Se	*P*val	Eaf	F
Garlic	rs11600909	A	G	-0.032	0.007	2.90E-06	0.076	21.9
Garlic	rs138637793	T	A	0.057	0.012	3.70E-06	0.023	21.4
Garlic	rs141575721	A	G	-0.041	0.008	6.70E-07	0.056	24.7
Garlic	rs143374250	A	G	0.060	0.013	2.20E-06	0.021	22.4
Garlic	rs147518355	C	A	0.068	0.014	2.10E-06	0.019	22.5
Garlic	rs149467747	A	G	0.090	0.019	3.30E-06	0.011	21.6
Garlic	rs17640594	A	C	0.092	0.020	3.80E-06	0.008	21.4
Garlic	rs2111374	C	T	0.040	0.008	2.40E-06	0.048	22.3
Garlic	rs2574953	G	A	-0.048	0.010	2.10E-06	0.967	22.5
Garlic	rs4584661	T	G	0.048	0.010	3.20E-06	0.031	21.7
Garlic	rs56269735	C	A	0.045	0.010	3.70E-06	0.035	21.4
Garlic	rs56317696	A	G	-0.018	0.004	8.00E-07	0.417	24.4
Garlic	rs60010968	A	T	0.045	0.010	3.50E-06	0.037	21.5
Garlic	rs62081074	A	T	-0.026	0.005	2.00E-06	0.129	22.6
Garlic	rs6835673	G	A	0.041	0.008	1.30E-06	0.049	23.4
Garlic	rs72780997	C	A	0.030	0.006	1.90E-06	0.089	22.7
Garlic	rs74679312	G	A	0.041	0.008	3.30E-07	0.056	26.1
Garlic	rs77374784	G	A	-0.033	0.007	4.60E-06	0.068	21.0
Garlic	rs78818759	T	C	0.068	0.014	6.30E-07	0.019	24.8
Onion	rs113181638	C	T	0.084	0.018	3.80E-06	0.023	21.4
Onion	rs117193414	C	T	0.099	0.019	4.00E-07	0.019	25.7
Onion	rs11961853	T	C	-0.034	0.007	1.60E-06	0.178	23.0
Onion	rs12464142	A	C	0.049	0.010	2.10E-06	0.070	22.5
Onion	rs144108770	T	C	0.086	0.018	2.40E-06	0.023	22.2
Onion	rs146199307	A	G	0.142	0.028	4.30E-07	0.011	25.5
Onion	rs1467978	C	A	-0.028	0.006	4.00E-07	0.368	25.7
Onion	rs188458079	C	T	0.180	0.034	1.30E-07	0.007	27.8
Onion	rs4378921	T	C	0.035	0.008	3.40E-06	0.145	21.6
Onion	rs6890703	T	C	-0.029	0.006	1.30E-06	0.307	23.4
Onion	rs7511986	A	G	0.027	0.006	3.80E-06	0.694	21.4
Onion	rs783494	C	T	0.025	0.005	4.80E-06	0.550	20.9
Onion	rs79181049	A	G	0.067	0.015	4.90E-06	0.035	20.9
Onion	rs9640159	G	T	0.025	0.005	4.00E-06	0.418	21.3

Beta = the per-allele effect on cannabis use, EA = effector allele, OA = other allele, Se = standard error, SNPs = single nucleotide polymorphisms.

### 3.2. Causal relationship garlic or onion on GC

**Figure 1.**
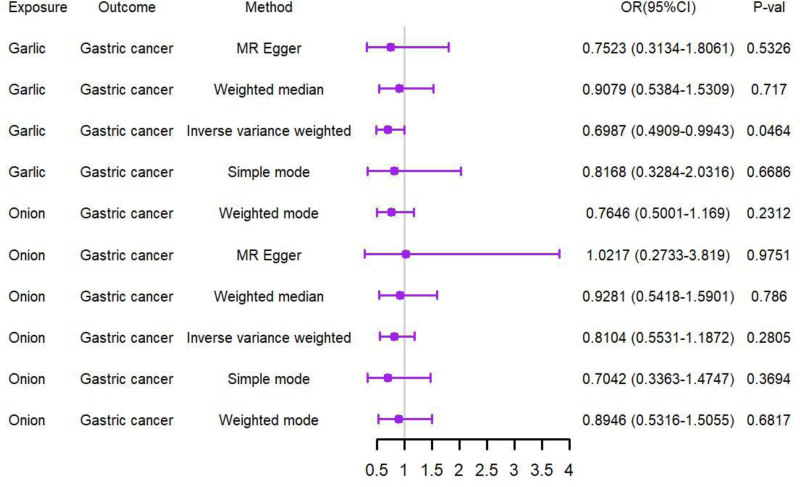
Forest plot of the causal effect of garlic or onion on GC MR analysis. GC = gastric cancer, MR = Mendelian randomization.

IVW analysis results showed that garlic was negatively significant associated with GC risk (odds ratio = 0.70; 95%CI 0.49–0.99; *P* = .046); but the *P*-values of the other 4 models are >.05. Onion was not associated with GC risk, and its *P*-value was higher than .05. The MR estimation results of garlic or onion SNPs and GC risk were presented in Table 3 and Figure 1.

**Table 3:** The garlic or onion on GC MR analysis results.

Exposure	Outcome	Methods	SNP (n)	Beta	*P*-value
Garlic	Gastric cancer	MR Egger	19	-0.2846	.5326
		Weighted median	19	-0.0966	.7170
		Inverse variance weighted	19	-0.3586	.0464
		Simple mode	19	-0.2023	.6686
		Weighted mode	19	-0.2684	.2312
Onion		MR Egger	14	0.0215	.9751
		Weighted median	14	-0.0746	.7860
		Inverse variance weighted	14	-0.2103	.2805
		Simple mode	14	-0.3507	.3694
		Weighted mode	14	-0.1114	.6817

Beta = the per-allele effect on cannabis use.

**Figure 2.**
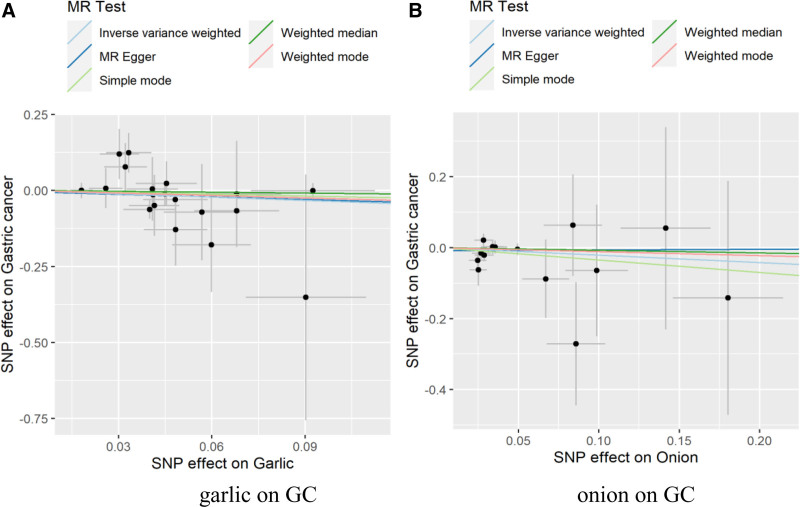
Scatter plots of MR analysis of relationships garlic or onion on GC. GC = gastric cancer, MR = Mendelian randomization.

**Figure 3.**
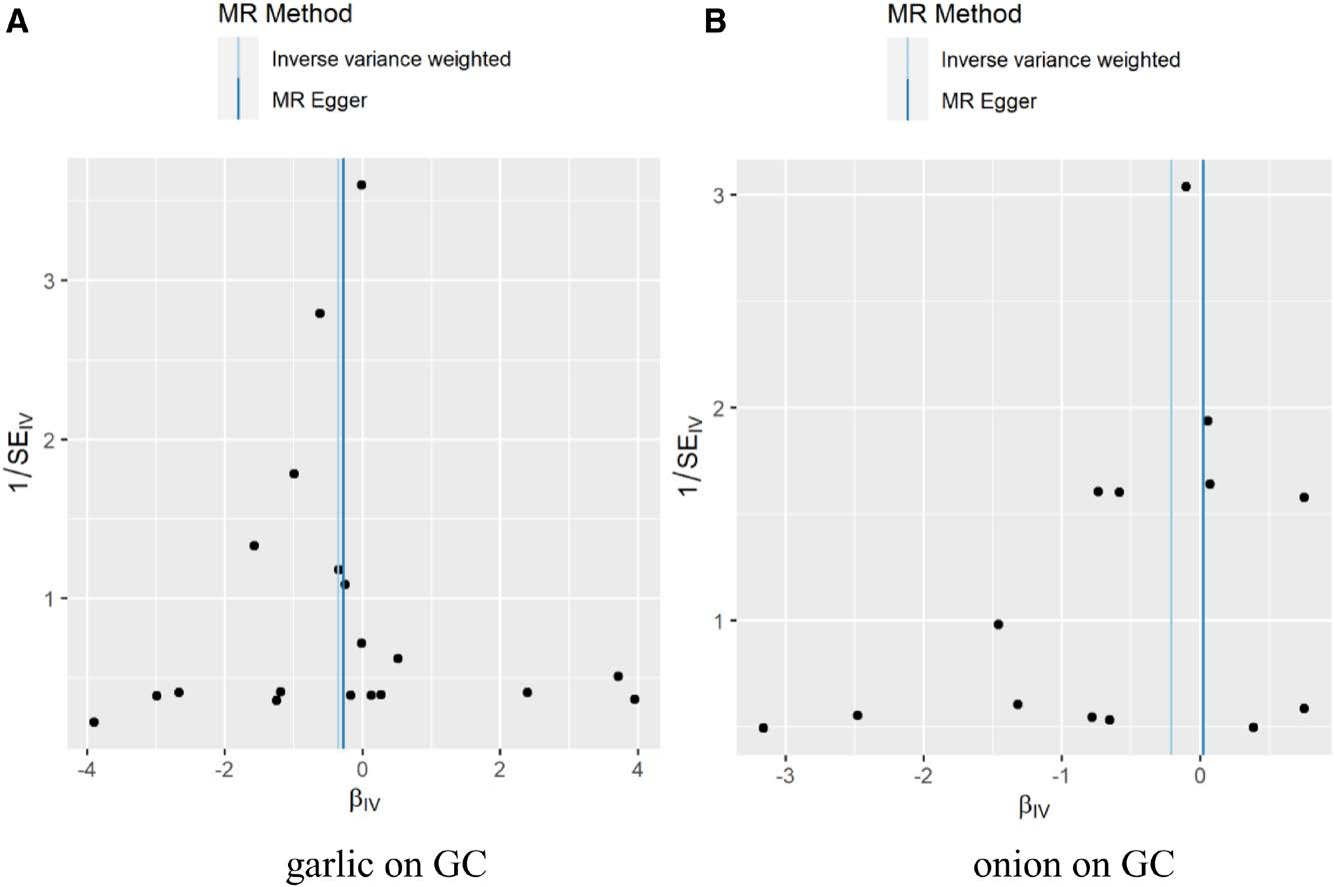
Funnel plots of MR analysis of relationships garlic or onion on GC. GC = gastric cancer, MR = Mendelian randomization.

**Figure 4.**
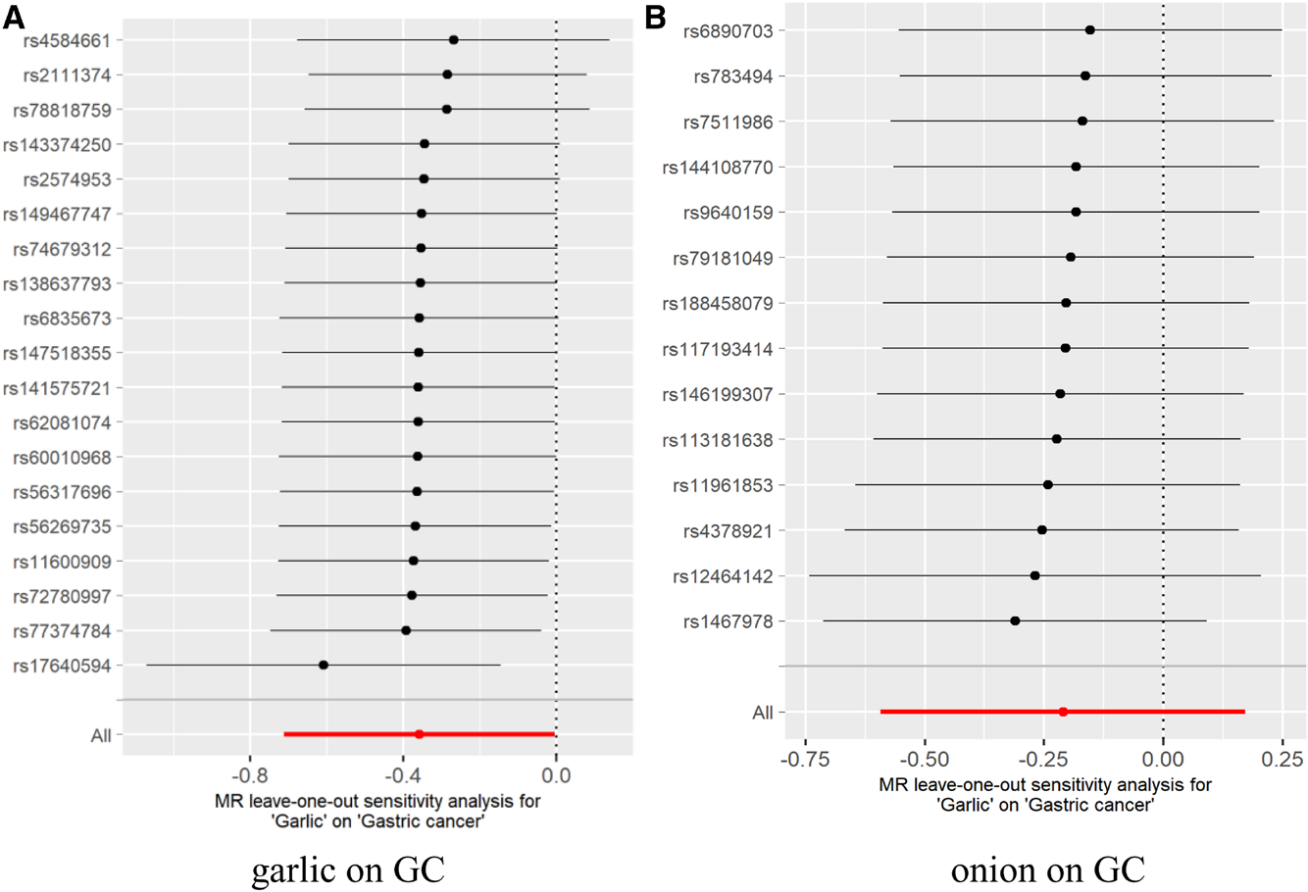
Leave-one-out plots of MR analysis of relationships garlic or onion on GC. GC = gastric cancer, MR = Mendelian randomization.

In the sensitivity analysis of the results, the Cochran *Q* test showed that there was no heterogeneity garlic on GC (Q_IVW_ = 17.30; *P*_IVW_ = .503; Q_MR-Egger_ = 17.26; *P*_MR-Egger_ = .437); there was no heterogeneity onion on GC (Q_IVW_ = 10.20; *P*_IVW_ = .677; Q_MR-Egger_ = 10.07; *P*_MR-Egger_ = .610). The horizontal pleiotropy test *P*-values were >.05, which indicated that there was no evidence of horizontal pleiotropy. The leave-one-out analysis results showed that the effect values of SNPs were all on the right side of 0, which indicated that SNPs had no effect on the overall effect estimation, and the overall results of MR analysis were relatively stable. The specific sensitivity analysis results are shown in Table 4, and Figures 2–4 were Scatter plots, funnel plots and leave-one-out plots of the garlic or onion on GC significant results.

**Table 4:** Evaluation results of heterogeneity and pleiotropy.

Exposure	Outcome	Pleiotropy test			Heterogeneity test			
		MR-Egger			MR-Egger		IVW	
		Intercept	SE	*P*	Q	Q_pval	Q	Q_pval
garlic	GC	-0.004	0.024	.858	17.264	0.437	17.298	0.503
onion	GC	-0.009	0.024	.725	10.074	0.61	10.203	0.677

Q_pval = The *P*-value of Cochran *Q* test.

## 4. Discussion

In this study, MR analysis results showed that eating more garlic was associated with a reduced risk of GC. However, no causal relationship was observed between onions and GC. In addition, sensitivity analysis indicates that there is no evidence of heterogeneity and horizontal pleiotropy in the results, and the results of MR analysis are not affected by any SNP. Therefore, the results of MR analysis are stable and reliable.

Garlic and onions are one of the oldest cultivated plants in the world, originating from Central Asia. Garlic and onions both have a spicy taste, and ancient medicine in multiple countries has recorded the application of onion vegetables in treating diseases.^[40]^ Scallion vegetables contain various bioactive sulfur-containing compounds, such as diallyl disulfide and diallyl trisulfide. Garlic and onions have antibacterial and antioxidant properties. Diallyl disulfides inhibits the proliferation of *H pylori* in culture, and garlic water extract can inhibit the proliferation of *H pylori*, which is associated with the risk of gastric cancer.^[41]^ Garlic and onions have anti-cancer properties, and their sulfur-containing compounds inhibit tumor growth through various pathways. For example, by inhibiting the mucosal transformation caused by N-nitroso compounds, tumors can be prevented^[42]^; Diallyl disulfides inhibits tumors through pathways such as RAF/MEK/ERK and mitochondrial dependence^[43]^; Ddiallyl trisulfides fights against tumors by blocking the cell cycle, inhibiting tumor cell proliferation, and inhibiting angiogenesis.^[44]^ Regulate the activity of metabolic enzymes that activate cytochrome P450 or detoxify carcinogens, and inhibit tumor growth.^[45]^ Garlic is rich in allicin, while scallions and onions contain limited amounts of allicin Allicin (diallyl thiosulfite) has a pungent odor.^[46]^ Allicin can undergo a thiol disulfide exchange reaction with protein thiols, which plays a crucial role in the biological activity of allicin.^[47]^ Allicin has anti-cancer properties and can inhibit the key risk factor of cancer–HP.^[48]^ Allicin fights against digestive system tumors by inhibiting tumor proliferation, invasion, and angiogenesis.^[49]^ Allicin exhibits antiproliferative potential on gastric adenocarcinoma cells.^[50]^ Allicin can not only prevent tumors, but also play a beneficial role in cancer treatment under certain conditions.^[51]^ The above are consistent with our hypothesis that garlic may be a potential solution for preventing and treating gastric cancer. Due to the low content of allicin in onions, it is inconsistent with our hypothesis, but consistent with the results of our MR analysis.

Multiple observational studies have found a certain negative association between onion vegetables and gastric cancer.^[27–30]^ Our research findings are consistent with the results of a 22.3-year randomized follow-up intervention trial of 144 GC event cases. Garlic vegetable intake is associated with a reduced risk of GC occurrence (*P* trend = .02; odds ratio: 0.83; 95% CI: 0.70–0.98), while onion intake is not correlated.^[29]^ The potential difference in the role of garlic and onions in gastric cancer may be due to differences in the amount and way people consume onion vegetables, in addition to the difference in the amount of allicin they contain. For example, garlic and onions have different consumption patterns. Some places eat onions raw, while others eat onions cooked. After cooking, the content of quercetin in onions decreases by 30%.^[52]^ Similarly, people from different regions choose to eat garlic raw or cooked, as heating can disrupt the formation of sulfur compounds in garlic.^[53]^ For example, the consumption of garlic and onions is different. In some places, onion consumption is higher, while in others, garlic consumption is higher. By increasing onion intake, the GC risk tends to decrease.^[54]^ However, it is worth noting that these results only apply to onions and garlic, and further research is needed on other vegetables in the Alliaceae family to gain a deeper understanding of the relationship between Alliaceae and GC.

This study has some advantages. Firstly, our study is based on publicly available summary data from GWAS, and the data source for this study is reliable. Secondly, we conducted sensitivity analysis to ensure the stability of the results. Finally, compared to observational studies, MR analysis is better because SNPs are randomly assigned and the bias in the analysis results is relatively small.

However, this study also has some limitations. Firstly, the results of this study are based on European ancestry, while GC patients are mainly concentrated in Southeast Asia. Therefore, it is currently unclear whether the results can be extended to Asian ancestry. To further explore the generalizability of our findings, future studies could include data from other populations with different genetic backgrounds and dietary habits. This would allow for a more comprehensive understanding of the causal relationship between garlic and onion intake and the risk of GC across different populations. Additionally, studies could investigate the potential differences in the role of garlic and onions in GC prevention and treatment among different populations, taking into account factors such as genetic variation, dietary patterns, and environmental exposures. Secondly, we acknowledge that dietary habits can change over time, and the genetic variants used in MR analysis reflect lifelong exposure. If garlic and onion consumption changes significantly over a person’s lifetime, the genetic proxies may not accurately represent current dietary habits. This could potentially affect the validity of our results.To deal with this limitation and validate our results, future studies could consider incorporating longitudinal data on dietary habits and genetic information. This would allow for a more accurate assessment of the relationship between garlic and onion intake and the risk of GC, taking into account changes in dietary habits over time.

## 5. Conclusions

This MR study provides genetic evidence to support the association between garlic and reduced risk of GC in European ancestry. However, no negative relationship was observed between onion and European ancestry GC. These findings provide insights into the prevention and treatment of GC. However, further large-scale clinical studies of different populations are needed in the future to validate these findings.

## Author contributions

**Conceptualization:** Zhaoyin Wang, Pengfei Liu, Jingbin Wang.

**Data curation:** Zhaoyin Wang, Pengfei Liu, Jingbin Wang.

**Formal analysis:** Zhaoyin Wang, Pengfei Liu.

**Investigation:** Pengfei Liu, Pengli Ma.

**Methodology:** Pengfei Liu.

**Project administration:** Zhaoyin Wang.

**Software:** Zhaoyin Wang, Pengfei Liu.

**Supervision:** Jingbin Wang, Pengli Ma.

**Validation:** Zhaoyin Wang.

**Writing – original draft:** Jingbin Wang, Pengli Ma, Xinyao Liu.

**Writing – review & editing:** Jingbin Wang, Pengli Ma.
